# Bank Account Ownership and Access Among In-Patients in Psychiatric Care in Berlin, Germany—A Cross-Sectional Patient Survey

**DOI:** 10.3389/fpsyt.2020.00508

**Published:** 2020-06-03

**Authors:** Stefanie Schreiter, Felix Bermpohl, Meryam Schouler-Ocak, Michael R. Krausz, Wulf Rössler, Andreas Heinz, Stefan Gutwinski

**Affiliations:** ^1^Charité – Universitätsmedizin Berlin, Corporate member of Freie Universität Berlin, Humboldt-Universität zu Berlin, and Department of Psychiatry and Psychotherapy, Berlin Institute of Health, Berlin, Germany; ^2^Department of Psychiatry and Psychotherapy, Psychiatrische Universitätsklinik der Charité im St. Hedwig-Krankenhaus, Berlin, Germany; ^3^Department of Psychiatry, University of British Columbia (UBC), Vancouver, BC, Canada; ^4^Klinik für Psychiatrie und Psychotherapie, Universität Zürich, Zürich, Switzerland

**Keywords:** bank account, financial exclusion, mental health, psychiatric in-patients, social exclusion

## Abstract

**Background:**

Access to a bank account is critical for overall participation in social life and an indicator for social integration. Worldwide about 1.7 billion people remain with no access to banking facilities as a form of financial exclusion which represents 31% of the world’s general population. In contrast, in Western countries like Germany, 99% of the general population use bank accounts.

**Methods:**

We conducted an exploratory cross-sectional survey on bank account ownership and bank account access among psychiatric in-patients in a psychiatric hospital in Berlin. Out of 540 participants who were reached for an interview, 486 shared information about bank account ownership and 469 on access.

**Results:**

Out of 486 participants 49 (10.1%) did not own a bank account. Among the remaining 420 participants owning a bank account, 36 (8.3%) did not have direct access to their bank account, but only, e.g., their legal guardian. Regression results found psychosis, intellectual disabilities, a longer treatment duration, as well as being of male gender and a more instable housing status to be significantly associated with a missing bank account or a missing access to one’s bank account.

**Conclusions:**

The lack of bank account ownership and access among this population of psychiatric patients is concerning. The interrelationship between factors of financial exclusion and mental health should be further explored in longitudinal studies. More attention is needed to support people with severe mental illness to be able to access resources associated with financial inclusion.

## Introduction

Globally, about 1.7 billion adults remain “unbanked”—without an account at a financial institution or through a mobile money provider (1). However, the ability to be in control of one’s finances is an essential part of participation in societies. Financial services help people escape from poverty by facilitating investments in their health, education, and businesses and simplify the management of financial emergencies, e.g., job loss (1). Additionally, in most countries, a bank account is necessary to rent a flat, which is, in turn, necessary to find a job. That is why the World Bank has made it a key priority to promote financial inclusion—access to and use of formal financial services. The Global Findex database shows that approximately 69% of adults worldwide have an account, up from 62% in 2014 and 51% in 2011. In high-income economies the rate of adults with a bank account is fairly higher, e.g., 99% of population in Germany, 93% in the US, 100% in Canada, and 96% in the UK have an account (1). Therefore, most unbanked adults worldwide live in the developing world (1).

Regarding aspects of mental health, authors like Taylor et al. (2) document a positive relationship between the individuals’ ability to manage and take control of their finances and their psychological wellbeing (2), since it increases financial stability, reduces stress, and benefits health. Moreover, studies suggest financial stress to have a strong and consistent relationship with the occurrence of common mental disorders (3).

In a study examining the degree to which observed differences in self-reported health status between African Americans, Asians, Latinos, and non-Hispanic Whites in the United States can be attributed to differences in various indicators of socioeconomic status, checking and savings account ownership did predict better self-reported health for Latinos (4). For older Hispanics in the United States, bank account ownership was associated with better mental health (5). A recent study in Ghana reported that older people with some level of financial engagement were more likely to report better self-rated health and lower levels of psycho-logical distress but were less likely to use health services (6). Thus, it seems that the individual’s ability to take control of their finances is a relevant factor for vulnerable or marginalized groups.

Since poverty and poor mental health are closely related (3), we explored financial inclusion in form of bank account access and ownership among users of the psychiatric healthcare system by a cross-sectional patient survey in one of the largest psychiatric hospitals in Berlin. The goal was a comprehensive description of bank account access and ownership and related clinical and socio-demographic factors.

## Methods

### Study Design and Participants

The study was part of the “WOHIN-project” (7), which is a cross-sectional survey, designed to investigate the housing situation, psychiatric morbidity and service use among psychiatric in-patients and day-clinic patients treated in the catchment area of the Psychiatric University Hospital Charité at St. Hedwig Hospital over a 6-months period (15th March to 15th September 2016). The hospital provides psychiatric treatment for all inhabitants living in the catchment area of Berlin’s districts Wedding, Tiergarten, and Moabit. These districts struggle with comparable problems of larger cities in Germany due to partially low living standards and high rates of migrants. The hospital offers in-patient treatment for 192 people spread out on three general psychiatric wards and four specialized wards [addiction, depression, geronto-psychiatry, “Soteria” (treatment of people with early psychosis)] as well as five day-clinics. In the study period, a total number of 1,251 patients were admitted (excluding out-patients and re-admissions).

Trained interviewers contacted patients as soon as possible after admission. All participants gave written informed consent before participation. The local ethics committee of the Charité Berlin approved the study (Number: EA1/291/15). A monetary incentive (5€) was offered for participation. 540 subjects (43.2%) were willing to participate in the interview, of which 486 gave information about bank account ownership and 469 on bank account access.

Socio-demographic variables were covered by the structured interview. Diagnoses of mental disorders were based on discharge records and provided by psychiatric clinicians based on ICD-10 criteria (8). Participants with psychosis underwent assessment of psychopathology measured by the Positive and Negative Syndrome Scale (PANSS) (9).

### Statistical Analysis

After a descriptive analysis, comparative analyses (Mann-Whitney UTest and Chi-square test) were performed in order to determine whether the relationship between having a bank account respectively having access to one’s bank account and the considered variables was significant. Due to the exploratory approach of the study, we did not perform a p-value correction for multiple testing in analyses of group differences and considered results to be significant p < 0.05. Afterward, the variables that showed a significant effect on bank account ownership respectively having access to one’s bank account were introduced in a multivariable binary logistic regression model in order to determine their significance in the association with the dependent variables (bank account ownership and the ability to access one’s bank account). We here performed Bonferroni correction. Data was analyzed with SPSS version 19.0 [IBM Corp, Armonk, NY, USA (10)].

## Results

Among 486 participants, 49 (10.1%) reported not to have their own bank account. Among the remaining 420 participants owning a bank account, 36 (8.6%) did not have access to their bank account. Among those participants who had access, 21 (5.5%) were not able to freely command over their money and were instead depending on a legal guardian, a family member, staff of their therapeutic institution, or had an impounded bank account.

Ten percent of the participants refused to give information about bank account ownership and further 17 on access; reasons for refusal were not collected. This sub-group did not differ significantly from participants giving information with regard to the main demographic variables (age: 44.13 vs. 42.31 years, t = 0.85; p = 0.394; male gender: 51.9% vs. 58.6%, X² = 0.90; p = 0.211, educational years: 14.95 vs. 14.21 years, t = 1.051; p = 0.294, form of income: X² = 1.54; p = 0.462, housing status: X² = 0.59; p = 0.900).

Results of Chi-square and Mann-Whitney U Test comparing sociodemographic and clinical variables between participants owning and participants not owning a bank account are presented in **Supplementary Table 1**. Participants with no bank account significantly differed from participants owning a bank account regarding being of male gender, fewer educational years, unstable housing status, depending on social benefits as income and earlier age of first psychiatric treatment (in- or out-patient) as well as a higher occurrence of the diagnoses of substance abuse, substance dependence, and intellectual disabilities.

Regarding bank account access, fewer educational years, unstable housing status, depending on social benefits as income and a diagnosis of intellectual disability were found significant in group-testing (**Supplementary Table 2**). Additionally, participants with no access to their bank account were significantly more often diagnosed with psychosis and less often with mood disorders (**Supplementary Table 2**). In contrast to results of bank account ownership, no significant group differences were observed for gender and substance use or dependence.

Since we found a significant group difference in participants with and without bank account access regarding the diagnosis of psychosis, we further explored group differences regarding psychopathology for participants with psychosis. We found no significant group differences regarding PANSS Scoring between participants with and without bank account ownership respective access [PANSS Positive: bank account: M = 16 (Q1–Q3 = 12–22); No bank account: M = 24 (Q1–Q3 = 17–26); n = 85; Z = −1.74; p = 0.082; bank account access: M = 16 (Q1–Q3 = 12–21); No bank account access: M = 16 (Q1–Q3 = 12–24); n = 72; Z = −0.20; p = 0.843; PANSS Negative: bank account: M = 15 (Q1–Q3 = 11–23); No bank account: M = 18 (Q1–Q3 = 12–30); n = 88; Z = −1.14; p = 0.255; bank account access: M = 14 (Q1–Q3 = 11–20); No bank account access: M = 16 (Q1–Q3 = 14–25); n = 73; Z = −1.28; p = 0.200].

All variables with significant differences between groups were included in multivariable binary logistic regression analyses in order to assess the significance of each variable to bank account ownership respective bank account access (**Tables 1** and **2**). The variable income was not included since almost all study subjects, who had no bank account or no access to it, depended on social benefits. Especially, female gender significantly increased the odds of bank account ownership. Being homeless compared to having an own apartment as well as a diagnosis of an intellectual disability significantly decreased the odds of having a bank account. **Table 2** shows the results of the multivariable binary logistic regression model for bank account access. Being homeless or living in a socio-therapeutic facility compared to having an own apartment as well as psychosis and a longer duration of treatment significantly decreased the odds of bank account access. More educational years significantly increased the odds of having access to one’s bank account, but did not reach significance after Bonferroni correction (p = 0.05).

**Table 1:** Predictors of owning a bank account: Multivariable binary logistic regression model.

Variables (n = 429)	Adjusted OR (95% CI)	p-value*
Gender (Male vs. Female)	4.86 (1.68–14.06)	0.006
Educational years	1.11 (1.01–1.23)	0.078
Housing status		
Own Apartment	1	
Socio-therapeutic facilities^a^	0.36 (0.14–0.96)	0.084
Homeless^a^	0.29 (0.11–0.77)	0.026
Accommodated with friends/family^a^	0.55 (0.17–1.80)	0.648
Any substance dependence	1.08 (0.49–2.36)	1
Any substance abuse	0.54 (0.24–1.21)	0.266
Intellectual disabilities	0.12 (0.02–0.76)	0.048
Age of first psychiatric treatment	1.03 (0.99–1.07)	0.226

* Adjusted p-value was calculated based on Bonferroni’s method.

a Compared to Own Apartment; Significance of the model: p < 0.001.

**Table 2:** Predictors of having access to your own bank account: Multivariable binary logistic regression model.

Variables (n = 343)	Adjusted OR (95% CI)	p-value*
Gender** (Male vs. Female)	1.27 (0.52–3.09)	1
Educational years	1.14 (1.02–1.27)	0.050
Housing status		
Own Apartment	1	
Socio-therapeutic facilities^a^	0.28 (0.10–0.76)	0.024
Homeless^a^	0.24 (0.08–0.72)	0.022
Psychosis	0.33 (0.13–0.82)	0.032
Mood disorders	1.42 (0.41–4.94)	1
Duration of treatment	0.93 (0.90–0.97)	< 0.001

* Adjusted p-value was calculated based on Bonferroni’s method.

** Although gender was not significant in group-testing,we included gender in regression analysis as a basis parameter. Excluding gender in regression analysis showed no difference in regard to significant results.

a Compared to Own Apartment; Significance of the model: p < 0.001.

Intellectual disabilities were excluded due to a small sample size as well as participants staying with friends or family.

## Discussion

To our knowledge, this is the first study investigating financial inclusion in form of bank account ownership among psychiatric patients. In our population of psychiatric in-patients, 10.1% did not have an own bank account. These results are in contrast to Germany’s general population of whom 99% have a bank account, leaving a gap of 9.1% between people with severe mental illness and the general population (1). Limited by the missing healthy control group in this study, it is not easily deductible that the proportion of 1% of Germany’s general population remaining unbanked mostly is explained by the consequences of mental illness, but the results of this study strongly hint toward this assumption. Additionally, among the remaining participants owning a bank account, 8.6% did not have access to their account and were, in turn, depending on family, friends, or a legal guardianship, leaving them partially financially excluded.

Regarding mental health status, psychosis, intellectual disabilities, and a longer treatment duration were significantly associated with a missing bank account ownership or a missing access to one’s bank account in regression results. For people with a more severe course of illness or struggles in their daily management due to mental health related factors especially regarding psychosis and intellectual disabilities, a financial caregiver might have been installed to avoid harm due to illness related risk-behavior, although we did not find any group differences in psychopathology in participants with psychosis regarding bank account status. Our study did not examine whether that limited access was still necessary. These results underline people with psychosis or intellectual disabilities as two vulnerable patient groups among which financial inclusion should be regularly kept in sight. Further regression results revealed male gender and housing status (being homeless or living in a socio-therapeutic facility) as the most relevant sociodemographic variables in predicting bank account ownership and access. Fewer educational years as an associated factor did not remain significant after Bonferroni correction, but was throughout significantly associated in group-testing.

Regarding education, these findings are in line with international studies reporting that unbanked adults are more likely to have low educational attainment (1). We found, that even 18% of participants living in socio-therapeutic facilities did not own a bank account, compared to 22.8% among homeless and 4.4% among stably housed participants. This underlines the close connection between financial access and housing situation. In order to open a bank account in Germany generally you need a certificate of a registered address in Germany and a passport for all non-EU applicants. This barrier might explain the high rate of participants without a bank account among homeless participants, although we did not find significant group differences concerning participants with a foreign nationality. In Germany, 77.5% of homeless people were diagnosed with a mental disorder according to a recent meta-analysis (11). Substance related disorders especially alcohol dependence were the most common mental health issues (11) in the meta-analysis. Underlining these results, participants in our sample with substance dependence, but also participants with a substance abuse did significantly more often not own a bank account. The effect on bank account status might be more associated with being homeless according to our regression results than having a substance use disorder. An early focus on financial inclusion in vulnerable groups of homeless people with substance abuse problems seems crucial.

Almost all participants without a bank account depended on social benefits. The association between financial exclusion and a marginalized housing and employment situation as well as an longer duration of treatment, underline the importance of psychosocial therapies for people with severe mental illness in forms of supported housing or supported employment to assist with the participation in social life and self-determination especially in vulnerable phases of life (12).

### Limitations

Our study did only include participants from a certain catchment area, in a relatively poor neighborhood. Nevertheless, this neighborhood does not differ from other more disadvantaged German cities, e.g., regarding rates of under-employment which is 11.4% in Berlin Mitte (which includes our catchment area), 14.8% in Gelsenkirchen, 14.3% in Duisburg, 14.6% in Essen, 9.9% in Magdeburg, etc. (13). Still, generalization of results is limited due to the general response rate of 43%, which is also caused by short stays, during which patients could not be contacted, and patients who were not able to consent [for detailed description, see Schreiter et al., 2019 (7)]. Among the study sample, 90% of participants provided information on bank account status. The remaining 10% of the participants did not differ in main demographic variables (age, gender, educational years, form of income, housing status) from other participants. Due to the cross-sectional nature of the study, causal conclusions cannot be drawn. In future longitudinal studies could give further insight in the relation between development of mental illness and stress related to poverty and associated exclusion.

## Data Availability Statement

The datasets for this article are not publicly available due to data security reasons (identifiable data). Requests to access the datasets should be directed to SS (stefanie.schreiter@charite.de).

## Ethics Statement

The studies involving human participants were reviewed and approved by Ethikkommission der Charité - Universitätsmedizin Berlin. The patients/participants provided their written informed consent to participate in this study.

## Author Contributions

SS and SG were responsible for drafting and revising the original study protocol; they were the chief investigators and had overall responsibility for management of the trial; they delivered the training to the interviewers. MS-O, AH, WR, and FB revised the original study protocol. FB, AH, and MS-O provided additional clinical supervision. SS and SG wrote the analysis plan and cleaned and analyzed the data under supervision from MK, WR, and FB. SS and SG wrote the first draft of the report and revised subsequent draft. All authors contributed to and approved the final report.

## Conflict of Interest

The authors declare that the research was conducted in the absence of any commercial or financial relationships that could be construed as a potential conflict of interest.
