# Supplementary material for: Bank Account Ownership and Access Among In-Patients in Psychiatric Care in Berlin, Germany—A Cross-Sectional Patient Survey
Source: Front Psychiatry. 2020 Jun 3;11:508. doi: 10.3389/fpsyt.2020.00508 (PMC7283455; doi:10.3389/fpsyt.2020.00508)
Supplement: Supplementary file 1 [file Table_1.docx]

**Online Supplementary Material**

**eTable 1** Differences in socio-demographic and clinical variables between participants owning and participants not owning a bank account

| N=486^a^ | Has bank account  N/Mean  (% or SD) | Has no bank account  N/Mean  (% or SD) | Statistics |
| --- | --- | --- | --- |
| Number of participants | 437 (89.9%) | 49 (10.1%) |  |
| Male^b^ | 245 (56.2%) | 39 (79.6%) | X²(1)=9.94; p=0.001* |
| Age | 42.7 (14.7) | 38.5 (13.5) | t=-1.92(484); p=0.056 |
| Educational years (Median Q1-Q3) | 14 (12-17) | 12 (9-13) | Z=-4.98; p<0.001* |
| Years in school (Median Q1-Q3) | 11 (10-13) | 10 (8-11) | Z=-4.02; p<0.001* |
| Years of education after school (Median Q1-Q3) | 3 (2-5) | 3 (0-3) | Z=-3.42; p=0.001* |
| Housing status |  |  | X²=27.98(3); p<0.001* |
| Own Apartment/property | 281 (65.2%) | 13 (27.1%) |  |
| Socio-therapeutic facilities | 64 (14.8%) | 14 (29.2%) |  |
| Homeless | 44 (10.2%) | 13 (27.1%) |  |
| Accommodated with friends/family | 42 (9.7%) | 8 (16.7%) |  |
| Income |  |  | X²=13.61(2); p=0.001* |
| Salary (from full- or part-time work, vocational training,  Federal Education and Trainings Assistance, Savings) | 91 (22.2%) | 1 (2.6%) |  |
| Social benefits | 283 (69.2%) | 37 (97.4%) |  |
| Pension | 35 (8.6%) | 0 (0%) |  |
| Foreign Nationality | 97 (22.2%) | 16 (32.7%) | X²(1)=2.70; p=0.076 |
| Psychiatric disorders |  |  |  |
| Organic mental disorders | 24 (5.5%) | 3 (6.1%) | X²(1)=0.03; p=0.526 |
| Psychosis | 119 (27.2%) | 12 (24.5%) | X²(1)=0.17; p=0.413 |
| Any substance dependence | 197 (45.1%) | 29 (59.2%) | X²(1)=3.52; p=0.042* |
| Any substance abuse | 81 (18.5%) | 15 (30.6%) | X²(1)=4.05; p=0.038* |
| Mood disorders | 133 (30.4%) | 12 (24.5%) | X²(1)=0.74; p=0.246 |
| Anxiety disorders | 19 (4.3%) | 0 (0.0%) | X²(1)=2.22; p=0.127 |
| Posttraumatic stress disorder | 9 (2.1%) | 0 (0.0%) | X²(1)=1.03; p=0.381 |
| Personality disorders | 83 (19.0%) | 13 (26.5%) | X²(1)=1.58; p=0.143 |
| Intellectual disabilities | 9 (2.1%) | 4 (8.2%) | X²(1)=6.31; p=0.033* |
| Number of psychiatric disorders (excluding nicotine addiction) (Median Q1-Q3) | 1 (1-2) | 1 (1-2) | Z=-1.23; p=0.221 |
| Age of first psychiatric treatment (Median Q1-Q3) | 27 (20-38) | 21 (17-35) | Z=-2.77; p=0.006* |
| Suicide Attempt | 141 (34.1%) | 12 (24.5%) | X²=(1)1.81; p=0.116 |

SD, Standard Deviation; * significant group difference; ^a^ n=54 missing; ^b^ 1 patient not included due to transgender sex; missing data were excluded when calculating group statistics.

**eTable 2** Differences in sociodemographic and clinical variables between participants having access and participants not having access to their bank account (excluding participants without an own bank account

| Do you have access to your bank account?  N=420^a^ | Yes  N/Mean  (% or SD) | | No  N/Mean  (% or SD) | Statistics | |
| --- | --- | --- | --- | --- | --- |
| Number of participants | 384 (91.4%) | | 36 (8.6%) |  | |
| Male^b^ | 216 (56.4%) | | 21 (58.3%) | X²(1)=0.05; p=0.483 | |
| Age (Median Q1-Q3) | 42.37 (±14.57) | | 46.22 (±14.13) | t(418)=1.52; p=0.129 | |
| Educational years (Median Q1-Q3) | 14.0 (12.0-17.0) | | 12.5 (9.9-15.3) | **Z=-3.18; p=0.001** | |
| Years in school (Median Q1-Q3) | 11 (10-13) | | 10 (8-12) | **Z=-2.88; p=0.004** | |
| Years of education after school (Median Q1-Q3) | 3 (1.5-5.0) | | 2 (0-4.0) | **Z=-2.66; p=0.008** | |
| Housing status^c^ |  | |  | **X²(3)=17.51; p=0.001** | |
| Own Apartment/property | 256 (67.5%) | | 16 (44.4%) |  | |
| Socio-therapeutic facilities | 50 (13.2%) | | 11 (30.6%) |  | |
| Homeless | 33 (8.7%) | | 8 (22.2%) |  | |
| Accommodated with friends/family | 40 (10.6%) | | 1 (2.8%) |  | |
| Income |  | |  | **X²(2)=10.80; p=0.005** | |
| Salary (from full- or part-time work, vocational training, Federal Education  and Trainings Assistance, Savings) | 87 (24.0%) | | 0 (0.0%) |  | |
| Social benefits | 248 (68.3%) | | 31 (88.6%) |  | |
| Pension | 28 (7.7%) | | 4 (11.4%) |  | |
| Foreign Nationality | 79 (20.6%) | | 11 (30.6%) | X²(1)=1.92; p=0.122 | |
| Psychiatric disorders |  | |  |  | |
| Organic mental disorders | 18 (5.2%) | | 4 (11.4%) | X²(1)=2.27; p=0.132 | |
| Psychosis | 90 (26.0%) | | 16 (45.7%) | **X²(1)=6.14; p=0.014** | |
| Any substance dependence | 165 (47.7%) | | 12 (34.3%) | X²(1)=2.30; p=0.090 | |
| Any substance abuse | 65 (18.8%) | | 8 (22.9%) | X²(1)=0.30; p=0.348 | |
| Mood disorders | 106 (30.6%) | | 4 (11.4%) | **X²(1)=5.71; p=0.010** | |
| Anxiety disorders | 15 (4.3%) | | 1 (2.9%) | X²(1)=0.17; p=0.557 | |
| Posttraumatic stress disorder | 7 (2.0%) | | 0 (0.0%) | X²(1)=0.72; p=0.507 | |
| Personality disorders | 66 (19.1%) | | 4 (11.4%) | X²(1)=1.24; p=0.191 | |
| Intellectual disabilities | 4 (1.2%) | | 4 (11.4%) | **X²(1)=16.32; p=0.003** | |
| Number of psychiatric disorders (excluding nicotine addiction) (Median Q1-Q3) | 1 (1-2) | | 1 (1-2) | Z=-0.40; p=0.688 | |
| Age of first psychiatric treatment (Median Q1-Q3) | 29.0 (20.3-39.0) | | 24.5 (19.0-28.8) | Z=-1.59; p=0.112 | |
| Duration of treatment (Median Q1-Q3) | 8 (3.0-17.0) | | 17 (9.8-26.3) | **Z=-3.55; p<0.001** | |
| Do you have free command over your money? Excluding participants without an own bank account and participants without access to their bank account) N=382^d^ | yes  361 (94.5%) | no  21 (5.5%) | | |  |

SD, Standard Deviation; ^a^ n=17 missing; ^b^ 1 patient not included due to transgender sex; ^c^ n=4 missing; ^d^ including legal guardianship, family, therapeutic institution, Impounded bank account
